# Anti-tumor activity and toxicokinetics analysis of MGAH22, an anti-HER2 monoclonal antibody with enhanced Fcγ receptor binding properties

**DOI:** 10.1186/bcr3069

**Published:** 2011-11-30

**Authors:** Jeffrey L Nordstrom, Sergey Gorlatov, Wenjun Zhang, Yinhua Yang, Ling Huang, Steve Burke, Hua Li, Valentina Ciccarone, Tengfei Zhang, Jeffrey Stavenhagen, Scott Koenig, Stanford J Stewart, Paul A Moore, Syd Johnson, Ezio Bonvini

**Affiliations:** 1 MacroGenics, Inc., 9640 Medical Center Drive, Rockville, MD 20850, USA; 2 H. Lundbeck A/S, Ottiliavej 9, Valby 2500, Denmark

## Abstract

**Introduction:**

Response to trastuzumab in metastatic breast cancer correlates with expression of the high binding variant (158V) of the activating Fcγ receptor IIIA (CD16A). We engineered MGAH22, a chimeric anti-HER2 monoclonal antibody with specificity and affinity similar to trastuzumab, with an Fc domain engineered for increased binding to both alleles of human CD16A.

**Methods:**

MGAH22 was compared to an identical anti-HER2 mAb except for a wild type Fc domain. Antibody-dependent cell cytotoxicity (ADCC) assays were performed with HER2-expressing cancer cells as targets and human PBMC or purified NK cells as effectors. Xenograft studies were conducted in mice with wild type murine FcγRs; in mice lacking murine CD16; or in mice lacking murine CD16 but transgenic for human CD16A-158F, the low-binding variant. The latter model reproduces the differential binding between wild type and the Fc-optimized mAb for human CD16A. The JIMT-1 human breast tumor line, derived from a patient that progressed on trastuzumab therapy, was used in these studies. Single and repeat dose toxicology studies with MGAH22 administered intravenously at high dose were conducted in cynomolgus monkeys.

**Results:**

The optimized Fc domain confers enhanced ADCC against all HER2-positive tumor cells tested, including cells resistant to trastuzumab's anti-proliferative activity or expressing low HER2 levels. The greatest improvement occurs with effector cells isolated from donors homozygous or heterozygous for CD16A-158F, the low-binding allele. MGAH22 demonstrates increased activity against HER2-expressing tumors in mice transgenic for human CD16A-158F. In single and repeat-dose toxicology studies in cynomolgus monkeys, a species with a HER2 expression pattern comparable to that in humans and Fcγ receptors that exhibit enhanced binding to the optimized Fc domain, MGAH22 was well tolerated at all doses tested (15-150 mg/kg) and exhibited pharmacokinetic parameters similar to that of other anti-HER2 antibodies. Induction of cytokine release by MGAH22 in vivo or in vitro was similar to that induced by the corresponding wild type mAb or trastuzumab.

**Conclusions:**

The data support the clinical development of MGAH22, which may have utility in patients with low HER2 expressing tumors or carrying the CD16A low-binding allele.

## Introduction

HER2, an overexpressed cell-surface oncoprotein that contributes to breast, gastric, and other cancers [1], is a validated therapeutic target, as evidenced by clinical success of the monoclonal antibody (mAb) trastuzumab [2-5]. Trastuzumab acts against HER2-positive tumors by multiple mechanisms, including receptor internalization, receptor 'shedding', direct anti-proliferative activity, antibody-dependent cell-mediated cytotoxicity (ADCC), and presentation of antigenic determinants of opsonized cells to antigen-presenting cells [6]. The latter mechanisms depend upon the interaction of the Fc domain of trastuzumab with Fc-gamma receptors (FcγRs) expressed by immune effector populations, such as natural killer (NK) cells or mononuclear phagocytes [7-10]. Polymorphic variants of certain activating FcγRs predict response duration to trastuzumab: patients homozygous for CD16A (FcγRIIIA) 158V allele or CD32A (FcγRIIA) 131H allele or both have longer progression-free survival than patients carrying the respective 158F or 131R alleles [11], which bind the Fc domain of immunoglobulin G 1 (IgG1), the main class of therapeutic mAbs, such as trastuzumab, with lower affinity than their allelic counterparts.

FcγR polymorphism influences the clinical response to several IgG1 mAbs other than trastuzumab. While the relationship between CD16A polymorphism and benefit is controversial for cetuximab [12-15], CD16-158V and CD32A-131H homozygosity appear to be associated with beneficial responses for rituximab and infliximab [16-18]. Furthermore, for an agonistic anti-death receptor antibody with intrinsic anti-tumor activity that is potentiated by FcγR interactions, effector cells expressing the higher-binding CD16A and CD32A variants supported substantially greater proapoptoptic activity [19]. CD16A-158V homozygotes represent 10% to 20% of the population worldwide, whereas CD32A-131H homozygotes represent approximately 25% of Caucasians or Africans and 50% to 60% of Asians [20,21]. Thus, FcγR genotypes most frequently associated with greater beneficial responses occur in a minority of the population. This provides a strong rationale for engineering the Fc domain of trastuzumab to better interact with low-binding alleles of activating FcγRs to expand, without regard to FcγR genotype, the benefit of treatment to patients.

MGAH22 is an Fc-engineered mAb designed for increased binding to both alleles of CD16A and preservation of the direct anti-proliferative activity of trastuzumab. Since trastuzumab activity is enhanced in mice genetically deficient for the inhibitory FcγR, CD32B (FcγRIIB) [7], a negative regulator of activation of monocytes, macrophages, and dendritic cells [22], the Fc domain of MGAH22 was also engineered for reduced CD32B binding. The optimized Fc domain confers enhanced ADCC activity against HER2-positive tumors, including low HER2 expressors, independently of the FcγR variant for the effector cells. MGAH22 is active *in vitro *and *in vivo *against a HER2-positive tumor line derived from a patient whose tumor progressed while on trastuzumab. Because changes in effector cell interactions could have safety implications, high-dose MGAH22 toxicology studies were conducted in cynomolgus monkeys, a relevant species, with no significant antibody-related safety findings.

The enhanced properties of MGAH22 suggest potential clinical utility in extending the benefit of anti-HER2 immunotherapy to patients independently of their CD16A allelic expression and to patients who, because of low HER2 expression levels, do not qualify for trastuzumab treatment as well as to patients whose tumors progress while on trastuzumab.

## Materials and methods

### Human tumor cell lines

Breast (MCF-7, ZR-75-1, and SKBR-3), gastric (N87), colon (HT-29), and bladder (SW780) lines were obtained from the American Type Culture Collection (Manassas, VA, USA), and JIMT-1 (breast) was obtained from DSMZ (Braunschweig, Germany). All cell lines were cultured in accordance with recommended specifications for fewer than 30 passages. The number of HER2-binding sites per cell and immunohistochemistry category (0 to 3+) were determined by flow cytometry (QuantiBRITE™ PE; BD Biosciences, San Jose, CA, USA) and HercepTest™ (Dako, Carpinteria, CA, USA): MCF-7 (14,000; 1+), HT-29 (25,000; 1+), SW780 (37,000; 1+), ZR-75-1 (52,000; 2+), JIMT-1 (79,000; 2+); N87 (270,000; 2+), and SKBR-3 (540,000; 3+).

### Antibodies

ch4D5 was generated by fusing synthetic sequences encoding light- and heavy-chain variable domains of 4D5, the murine precursor of trastuzumab [23], to human κ and IgG1 constant domains, respectively. RES120 was generated from ch4D5 by light-chain mutagenesis (N65S) to eliminate a consensus N-glycosylation site. MGAH22 was generated from RES120 by exchanging its Fc domain for MGFc0264 (L235V, F243L, R292P, Y300L, and P396L) [24,25]. ch4D5-N297Q, which contains an inactivated Fc domain, was derived from ch4D5 by mutating the heavy-chain N-glycosylation site.

### Fc-gamma receptor binding

Binding of soluble forms of FcγRs (either monomeric extracellular domains or dimeric inactivated Fc-G2 fusions) to Fc domains was analyzed by surface plasmon resonance after capture of antibodies to immobilized HER2 [25].

### *In vitro *anti-proliferation activity

Tumor cells (2 × 10^4 ^per well) were incubated for 6 days with antibodies at 37°C, and proliferation/viability was detected by using the CellTiterGlo Luminescent Cell Viability Assay Kit (Promega Corporation, Madison, WI, USA).

### Antibody-dependent cell-mediated cytotoxicity

Peripheral blood mononuclear cells (PBMCs) were isolated from healthy human donor blood (Ficoll-Paque™ Plus; GE Healthcare, Piscataway, NJ, USA). NK cells were purified (Untouched Human NK cell isolation kit; Dynal, Invitrogen Corporation, Carlsbad, CA, USA). Target cells (2 × 10^4 ^per well) were incubated with antibodies for 30 minutes at 37°C in RPMI-1640 (without phenol red), 10% fetal bovine serum, and 2 mM GlutaMax™ (Invitrogen Corporation) before adding effector cells at an effector/target ratio of 30:1 (PBMCs) or 3:1 (purified NK cells). Lactate dehydrogenase release (Promega Corporation) was measured after overnight incubation. Cytotoxicity (expressed as a percentage) = (experimental cell lysis-antibody-independent cell cytolysis)/(maximum target lysis-spontaneous target lysis) × 100. FcγR genotypes were determined by sequencing polymerase chain reaction-amplified DNA.

### Treatment of xenograft tumors in mice

All mouse experiments were performed at our facility under protocols approved by the MacroGenics Institutional Animal Care and Use Committee. RAG2^-/- ^Balb/c mice (WT FcγR mice) were obtained from Taconic (Rockville, MD, USA). mCD16^-/- ^RAG2^-/- ^Balb/c mice (mCD16 knockouts) and mCD16^-/- ^hCD16A^+ ^RAG2^-/- ^mice (mCD16 knockouts transgenic for hCD16A-158F) were bred at MacroGenics. JIMT-1 cells (5 × 10^6 ^per mouse) in phosphate-buffered saline plus Matrigel were implanted subcutaneously and antibodies administered intraperitoneally weekly beginning at the time of tumor implantation or after tumors of approximately 200 mm^3 ^had been allowed to form. Tumor sizes were monitored three times weekly by orthogonal measurements with electronic calipers. Statistical differences in tumor sizes were assessed by two-way analyses of variance and Bonferroni post-test analyses (GraphPad Prism 5.02; GraphPad Software, Inc., La Jolla, CA, USA).

### Toxicology/toxicokinetics in non-human primates

Cynomolgus monkey experiments were conducted at Charles River Laboratories (Sparks, NV, USA) in accordance with Testing Facility Standard Operating Procedure, which adheres to the regulations outlined in the US Department of Agriculture Animal Welfare Act [26] and the conditions specified in the *Guide for the Care and Use of Laboratory Animals *[27]. The study protocols were approved by the Testing Facility Institutional Animal Care and Use Committee. A single-dose study was conducted with 12 cynomolgus monkeys randomly assigned to two groups (three per sex per group) receiving MGAH22 or RES120 at 50 mg/kg by 60-minute intravenous infusion. All animals were euthanized at day 62 for necropsies. A repeat-dose study was conducted with 40 monkeys randomly assigned to four groups (five per sex per group) receiving vehicle or MGAH22 weekly for 6 weeks at 15, 50, or 150 mg/kg by 60-minute intravenous infusion. Twenty-four (three per sex per group) were euthanized on day 40, 4 days after the last dose, and 16 (two per sex per group) were followed for a recovery period of 56 days and euthanized on day 93 for necropsies.

### Measurement of serum MGAH22 concentration by enzyme-linked immunosorbent assay

Goat anti-MGAH22-Fv antibody was used to capture MGAH22 (or RES120) from cynomolgus serum and this was followed by detection with biotinylated goat anti-MGAH22-Fv antibody plus streptavidin-alkaline phosphatase conjugate and 4-methylumbelliferyl phosphate as substrate. Product was measured by using a fluorescent microplate reader against a standard curve (four-parameter non-linear curve fitting). The minimum quantifiable concentration was 4.25 ng/mL.

### Measurement of cytokine release

BD™ human Th1/Th2 cytometric bead array (CBA) and Human IL-5 Flex Set CBA were used to measure levels of IL-2, IL-4, IL-5, IL-6, IL-10, tumor necrosis factor-alpha (TNF-α), and interferon-gamma (IFN-γ) in serum collected at different times after intravenous administration of antibodies to cynomolgus monkeys. Statistical differences in relative changes from baseline were assessed by non-parametric van Elteren tests (an extension to the Wilcoxon rank-sum test) and Bonferroni adjustments for multiple comparisons (SAS 9.2; SAS Institute Inc., Cary, NC, USA). Cytokine levels were also measured in cell culture supernatants after incubation of human PBMCs with antibodies on plates left uncoated or coated with 1 μg/mL of HER2 antigen-recombinant human ErbB2/HER2 Fc chimera (R&D Systems, Inc., Minneapolis, MN, USA), enzymatically deglycosylated-for 16 to 20 hours at 37°C. Statistical differences in cytokine levels induced by the different antibodies were assessed by a Wilcoxon signed rank test (SAS 9.2).

## Results

### MGAH22 preserves HER2-binding and anti-proliferative properties of trastuzumab

**Figure 1:**
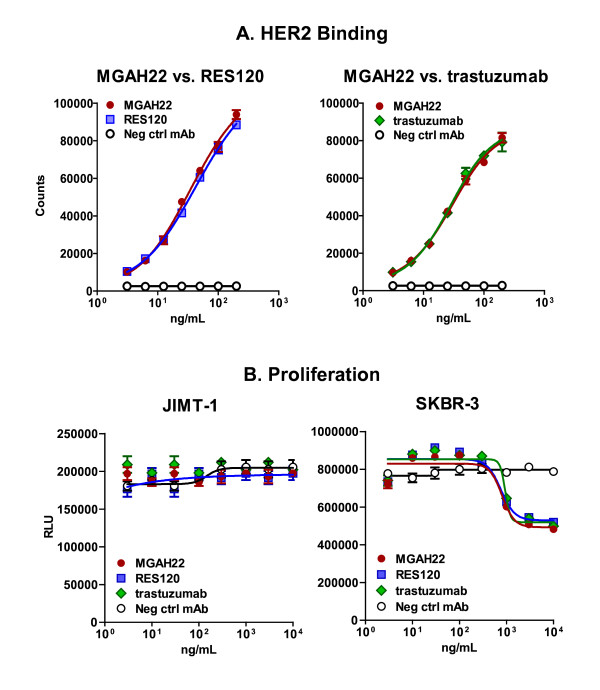
**HER2-binding and anti-proliferative activity**. **(a) **HER2-binding activity of MGAH22 was compared with RES120 or trastuzumab by antigen-capture enzyme-linked immunosorbent assay. EC_50 _(and 95% confidence interval) values were 39.33 (29.45 to 52.52) ng/mL for MGAH22 and 45.75 (33.37 to 62.67) ng/mL for RES120 (left panel) and 28.76 (24.96 to 33.15) for MGAH22 and 27.28 (23.6 to 31.53) ng/mL for trastuzumab (right panel). **(b) **Proliferation of JIMT-1 and SKBR-3 cells in the presence of MGAH22, RES120, or trastuzumab. Data are presented as mean ± standard error of the mean. EC_50_, effective concentration for 50% binding; MGAH22, chimeric anti-HER2 monoclonal antibody with an optimized Fc domain; Neg ctrl mAb, negative control monoclonal antibody; RES120, chimeric anti-HER2 monoclonal antibody with wild-type human immunoglobulin G 1 Fc domain; RLU, relative light units.

MGAH22 is a human/mouse chimeric IgG1 anti-HER2 antibody based on mouse clone 4D5, the precursor of trastuzumab [23] with an engineered Fc domain (MGFc0264) similar to the previously described Fc variant 18 [25], except that the V305I mutation was replaced by L235V to reduce CD32B binding. RES120, a molecule identical to MGAH22 except for a wild-type (WT) human IgG1 Fc domain, was used for comparison and as a trastuzumab surrogate. MGAH22 preserves the HER2-binding properties of RES120 and authentic trastuzumab (Figure 1a). Proliferation of SKBR-3 cells is sensitive to trastuzumab [23], whereas proliferation of JIMT-1 cells, a line with HER2 gene amplification but moderate expression and derived from a breast cancer patient whose metastatic tumor had progressed while on trastuzumab, is insensitive to trastuzumab [28]. MGAH22, RES120, and trastuzumab exhibit anti-proliferative activity indistinguishable from each other against SKBR-3 cells, but none shows activity against JIMT-1 cells (Figure 1b). Thus, the Fc domain modifications of MGAH22 do not influence the antigen recognition and anti-proliferative activity in the absence of effector cells.

### Binding of the optimized Fc domain to Fc-gamma receptors

**Figure 2:**
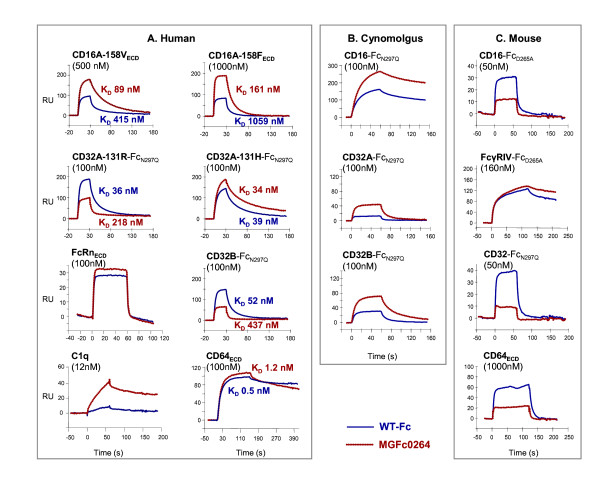
**Binding of monoclonal antibodies with wild-type or optimized Fc domains to FcγRs**. Representative surface plasmon resonance traces for binding of fixed concentrations of soluble human FcγRs or C1q **(a)**, cynomolgus monkey FcγRs **(b)**, or murine FcγRs **(c) **to ch4D5 (contains wild-type-Fc domain) or ch4D5-0264 (contains MGFc0264) captured on immobilized recombinant human HER2 protein are shown. hCD16A-158V, hCD16A-158F, and hCD64 were analyzed as soluble monomeric extracellular domains (ECDs), whereas hCD32A-131R, hCD32A-131H, and hCD32B were analyzed as soluble dimeric extracellular domain Fc-fusions (Fc_N297Q _or Fc_D265A_). Values of the equilibrium dissociation constant (K_D_) from full-range titration studies were determined by the fitting of equilibrium responses to a steady-state affinity model for hCD16 and hCD32 receptors or by a global fit to 1:1 binding model for hCD64 interactions that did not reach a steady state. FcγR, Fc-gamma receptor; RU, relative units.

Binding profiles of soluble FcγRs to mAbs with WT or optimized Fc domains were determined (Figure 2). Compared with the WT Fc domain, the optimized MGFc0264 domain demonstrates increased affinity for both alleles of human CD16A (equilibrium dissociation constant (K_D_) for 158V: from 415 nM to 89 nM; K_D _for 158F: from 1,059 nM to 161 nM) but decreased binding to human CD32B, inhibitory FcγR (K_D_: from 52 nM to 437 nM). It similarly imparts decreased binding to the 131R allele of human activating FcγR, CD32A (K_D_: from 36 nM to 218 nM), but binding to the 131H allele is not substantially modified (K_D_: from 39 nM to 34 nM). Owing to the substantially low affinity of human CD32A/B, K_D _values were obtained for the bivalent Fc fusion versions of these receptors (whose Fc domain was inactivated via N297Q or D265A mutation to eliminate homologous interactions); this strategy provides adequate sensitivity, due to avidity effects, to determine binding differences. The optimized Fc domain shows increased binding to human C1q. Binding to human CD64 (the high-affinity activating FcγR) and FcRn (the neonatal FcγR) is not substantially modified.

Compared with either allele of human CD16A, the WT human Fc domain exhibits increased binding to cynomolgus monkey CD16A, which has an invariant isoleucine at position 158 [29]. The optimized Fc domain imparts further increases in binding to monkey CD16A and CD32A (Figure 2b). In contrast to the decreased binding observed with human CD32B, MGFc0264 shows increased binding to monkey CD32B and this is most likely attributable to differences at position 131 (R in human and H in cynomolgus).

Murine CD16 is a low-affinity activating FcγR that is approximately 60% homologous to human CD32A but that, similarly to human CD16A, is distributed on murine NK cells and mononuclear phagocytes [22]. Binding of the optimized Fc domain of MGAH22 to murine CD16 is reduced (Figure 2c) and this contrasts to its increased binding to human or cynomolgus monkey CD16A. As with human CD32B, the optimized Fc domain imparts reduced binding to CD32, the mouse inhibitory FcγR (mice lack a CD32A ortholog), and reduced binding to murine CD64 and this contrasts with the minimal change observed with human CD64. Both WT and optimized human Fc domains bind similarly to mouse FcγRIV, although MGAH22 shows slightly improved binding (slower off-rate). FcγRIV is another low-affinity activating FcγR most closely related to human CD16A in sequence, but to human CD32A in distribution, because expression is restricted to murine myeloid cells and is absent on NK cells; furthermore, data suggest a potential functional relationship to human FcεRI [22,30,31].

### MGAH22 enhances the antibody-dependent cell-mediated cytotoxicity activity of effector cells expressing the CD16A-158F variant

**Figure 3:**
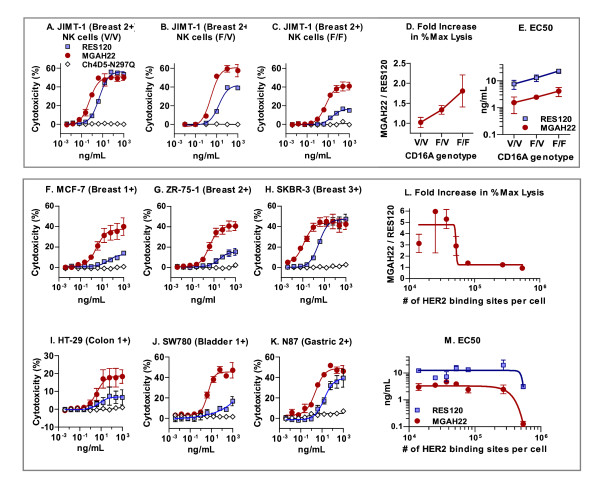
***In vitro *antibody-dependent cell-mediated cytotoxicity (ADCC) activity**. **(a-e) **ADCC with JIMT-1 as target cells and purified natural killer (NK) cells from two or three independent donors for each CD16A genotype (F/F, F/V, or V/V) as effectors using a 3:1 effector/target ratio. **(f-m) **ADCC with breast and non-breast cancer cell lines expressing HER2 at different levels as target cells and peripheral blood mononuclear cells from three to six independent CD16A-158F carriers as effectors using a 30:1 effector/target ratio. Fold increases in percentage maximal lysis by MGAH22 relative to RES120 and EC_50 _values are plotted according to CD16A genotype of effectors (d, e) or number of HER2-binding sites per target cell (l, m). Data are presented as mean ± standard error of the mean. EC_50_, effective concentration for 50% lysis; MGAH22, chimeric anti-HER2 monoclonal antibody with an optimized Fc domain; RES120, chimeric anti-HER2 monoclonal antibody with wild-type human immunoglobulin G 1 Fc domain.

For ADCC assays, effector cells were isolated from human donors of different CD16A genotypes. Compared with RES120, MGAH22 showed increased maximum lysis and lower effective concentration for 50% lysis (EC_50_) with CD16A F/F or V/F effector cells (purified NK cells) against JIMT-1 breast cancer cells (Figure 3a-e). With CD16A V/V effector cells, MGAH22 exhibited lower EC_50 _but was unaccompanied by enhanced maximum lysis. In these experiments, ch4D5-N297Q was inactive, confirming the requirement for a functional Fc domain.

### MGAH22 mediates efficient antibody-dependent cell-mediated cytotoxicity against tumor cells expressing low HER2 levels

MGAH22 demonstrated ADCC activity with EC_50 _values lower than those of RES120 against breast and non-breast cancer cell lines encompassing a wide range of surface HER2 expression with effector cells (PBMCs) from CD16A F/F or V/F donors (Figure 3f-m). No activity occurred when a HER2-negative breast cancer cell line (MDA-MB-468) [32] was tested (data not shown). The increase in maximum lysis with MGAH22 was inversely proportional to HER2 levels, and the greatest enhancement occurred with cells expressing lower HER2 densities. At very high HER2 levels, such as with SKBR-3 target cells, no increase in maximum lysis was observed with MGAH22; however, a substantial decrease in EC_50 _was still observed.

### Increased anti-tumor activity of MGAH22 in mice transgenic for human CD16A-158F

While mice bearing human tumor xenografts are commonly used to demonstrate anti-tumor activity of human IgG1 mAbs, mouse FcγRs are distributed and bind human IgG1 differently than their human counterparts (compare Figures 2a and 2c). This hampers the utilization of mouse xenograft models to assess the functional properties of mAbs optimized for human FcγR interactions. To ascertain the role of FcγRs in MGAH22 anti-tumor activity, xenograft studies employed three mouse strains: FcγR-WT mice (WT murine FcγR repertoire), mCD16^-/- ^mice (lacking murine CD16), and mCD16^-/- ^hCD16A^+ ^mice (lacking mCD16 but transgenic for human CD16A-158F, the low-binding allele). In this transgenic mouse strain, human CD16A-158F is expressed by NK cells and mononuclear phagocytes [25], similarly to its cell type-specific expression in humans [33]. Although mFcγRIV expression is preserved, this transgenic mouse model effectively eliminates the confounding factor caused by differential binding to mCD16.

**Figure 4:**
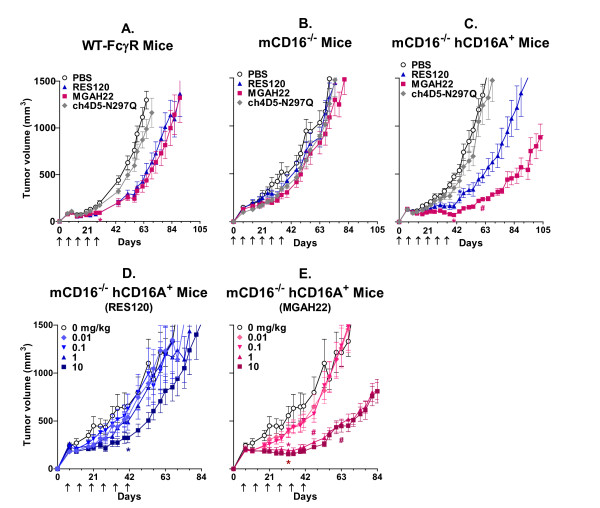
***In vivo *efficacy**. **(a) **Wild-type FcγR mice (10 per group): JIMT-1 cells implanted subcutaneously (s.c.) and monoclonal antibodies (mAbs) at 4 mg/kg administered five times at weekly intervals beginning at day 0. The first day of significant reduction in tumor size occurred at day 30 for both MGAH22 (*P *< 0.01) and RES120 (*P *< 0.01) compared with phosphate-buffered saline (PBS). **(b) **mCD16^-/- ^mice (11 per group): JIMT-1 cells implanted s.c. and mAbs at 2 mg/kg administered six times at weekly intervals beginning at day 0. **(c) **mCD16^-/- ^hCD16A^+ ^mice (8 per group): JIMT-1 cells implanted s.c. and mAbs at 2 mg/kg administered six times at weekly intervals beginning at day 0. The first day of significant reduction in tumor sizes occurred at day 37 for MGAH22 compared with PBS (*P *< 0.001), at day 44 for RES120 compared with PBS (*P *< 0.01), and at day 61 for MGAH22 compared with RES120 (*P *< 0.05). **(d, e) **mCD16^-/- ^hCD16A^+ ^mice (10 per group): JIMT-1 cells implanted s.c. and tumors of approximately 200 mm^3 ^allowed to form; RES120 (d) or MGAH22 (e) administered six times at weekly intervals beginning at day 6 at 0.01, 0.1, 1, or 10 mg/kg. The first day of significant reduction in tumor size occurred at day 32 for 1 or 10 mg/kg MGAH22 compared with PBS (*P *< 0.01), at day 41 for 10 mg/kg RES120 compared with PBS, at day 47 for 1 mg/kg MGAH22 compared with 1 mg/kg RES120 (*P *< 0.05), and at day 63 for 10 mg/kg MGAH22 compared with 10 mg/kg RES120 (*P *< 0.01). Data are presented as mean ± standard error of the mean. The first days of significant reduction in tumor sizes are indicated for MGAH22 or RES120 compared with vehicle (*) and for MGAH22 compared with RES120 (#). MGAH22, chimeric anti-HER2 monoclonal antibody with an optimized Fc domain; RES120, chimeric anti-HER2 monoclonal antibody with wild-type human immunoglobulin G 1 Fc domain.

JIMT-1 cells, which are insensitive to direct inhibition (Figure 1b), were selected for xenograft studies to eliminate confounding components pertaining to the anti-proliferative activity of anti-HER2 mAbs. This was confirmed by treating FcγR-WT mice bearing JIMT-1 xenografts with the Fc domain inactive ch4D5-N297Q mAb, which showed no anti-tumor activity (Figure 4a), indicating that only Fc-dependent mechanisms mediate activity against these xenografts. When MGAH22 or RES120 was administered to the same xenograft-bearing FcγR-WT mice, tumor growth was modestly inhibited, and the two antibodies were equally active. Administration of either mAb to mCD16^-/- ^tumor-bearing mice resulted in no anti-tumor activity (Figure 4b), and this suggests that mFcγRIV, in spite of its ability to bind to WT and optimized Fc domains, plays no role in this tumor model, possibly because of its lack of expression on NK cells and its restricted expression to myeloid cells.

When administered to JIMT-1 xenograft-bearing mCD16^-/- ^hCD16A^+ ^mice, MGAH22 exhibited enhanced anti-tumor activity compared with RES120 (Figure 4c), an activity attributable to the improved interaction between its optimized Fc domain and the human CD16A-158F (low-binding allele) receptor. In mCD16^-/- ^hCD16A^+ ^mice with established JIMT-1 tumors, MGAH22 exhibited significant anti-tumor activity at weekly doses of 1 or 10 mg/kg (Figure 4e), whereas RES120 exhibited only marginal anti-tumor activity at 10 mg/kg and no activity at 1 mg/kg (Figure 4d). These data support the hypothesis that MGAH22 is more active than an anti-HER2 mAb with a WT Fc domain, such as trastuzumab.

### Pharmacokinetics in cynomolgus monkeys

**Figure 5:**
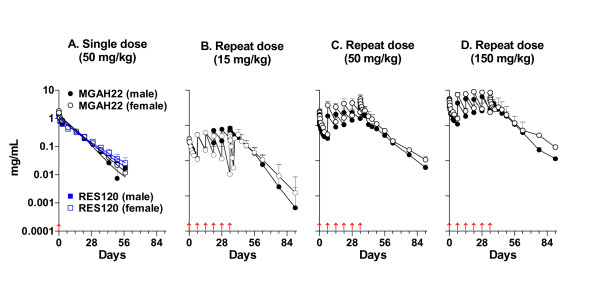
**Pharmacokinetics in cynomolgus monkeys**. Serum concentration-versus-time profiles for intravenous administration of MGAH22 or RES120 at a single 50 mg/kg dose **(a) **or MGAH22 at 15 **(b)**, 50 **(c)**, or 150 **(d) **mg/kg weekly for 6 weeks. Data are presented as mean ± standard error of the mean. MGAH22, chimeric anti-HER2 monoclonal antibody with an optimized Fc domain; RES120, chimeric anti-HER2 monoclonal antibody with wild-type human immunoglobulin G 1 Fc domain.

Pharmacokinetics of MGAH22 and RES120 were compared after single or repeated administrations in cynomolgus monkeys. Serum concentration-versus-time curves after a single dose of 50 mg/kg were biphasic, and pharmacokinetic parameters, calculated using a two-compartment elimination/distribution model, were similar for the two antibodies in males and females, except that the terminal elimination half-life of MGAH22 was approximately 20% shorter than that of RES120 (9.3 to 9.7 versus 11.7 to 12 days) (Figure 5 and Table 1). Two of six animals that received MGAH22 exhibited a rapid decline in serum concentration at days 22 to 29 and tested positive for anti-drug antibodies (ADAs), whereas none of the six animals that received RES120 were ADA-positive. In the repeat-dose study, MGAH22 was administered weekly for 6 weeks at doses of 15, 50, or 150 mg/kg, and animals in the recovery groups were followed for 56 days after the last dose. Values of C_max _(maximal serum concentration) and AUC_0-∞ _(area under the curve from zero to infinity) following the first dose appeared to increase linearly but were not dose-proportional after the sixth dose. Clearance was more rapid after the first dose than the sixth dose, and this likely reflects saturation of binding to target receptors. Terminal serum half-life was 7.3 to 8.9 days. One of the four animals in the low-dose recovery group exhibited a rapid decline in serum MGAH22 at the last few time points and tested positive for ADA at 56 days after the last dose, whereas ADA was not observed in any of the eight animals in the two higher-dose recovery groups; thus, incidence of ADA against the Fc-engineered molecule was not common in monkeys receiving multiple doses. Overall, the pharmacokinetic parameters for MGAH22 in cynomolgus monkeys are consistent with those of other anti-HER2 mAbs [34,35].

**Table 1:** Pharmacokinetic parameters for MGAH22 and RES120 in cynomolgus monkeys

Study	Monoclonal antibody	Dose, mg/kg	Sex	**C**_**max**_**, mg/mL**	**AUC**_**0-¥**_**, mg-hour/mL**	**T**_**1/2b**_**, days**	Clearance, mL/hour	**V**_**ss**_**, mL**
Single-dose	RES120	50	Male	1.40 ± 0.32	285.7 ± 4.2	11.7 ± 2.4	0.44 ± 0.01	170 ± 36
			Female	1.43 ± 0.05	311.8 ± 54.6	12.0 ± 2.3	0.41 ± 0.07	158 ± 15
	MGAH22	50	Male	1.62 ± 0.10	294.1 ± 53.2	9.3 ± 1.8	0.43 ± 0.07	132 ± 2
			Female	1.70 ± 0.14	314.2 ± 31.3	9.7 ± 1.1	0.40 ± 0.04	127 ± 8
Repeat-dose	MGAH22 (first dose)	15	Male	0.43 ± 0.06	57.0 ± 11.2	5.6 ± 2.0	0.82 ± 0.18	148 ± 35
			Female	0.43 ± 0.40	42.8 ± 9.7	5.1 ± 1.2	1.09 ± 0.24	144 ± 27
		50	Male	1.37 ± 0.23	205.7 ± 127.0	6.9 ± 5.0	0.82 ± 0.28	161 ± 24
			Female	2.85 ± 1.37	286.6 ± 98.2	6.6 ± 4.8	0.57 ± 0.21	109 ± 46
		150	Male	4.10 ± 0.49	558.1 ± 168.6	7.3 ± 3.7	0.78 ± 0.23	176 ± 60
			Female	6.22 ± 1.44	882.9 ± 347.7	7.9 ± 3.6	0.55 ± 0.32	127 ± 34
	MGAH22 (sixth dose)	15	Male	0.89 ± 0.11	229.8 ± 82.1	8.9 ± 4.3	0.24 ± 0.12	66 ± 6
			Female	0.98 ± 0.33	209.4 ± 110.4	8.7 ± 4.6	0.29 ± 0.16	90 ± 51
		50	Male	4.90 ± 5.34	975.5 ± 747.1	8.8 ± 3.0	0.22 ± 0.13	65 ± 34
			Female	7.20 ± 5.70	1,040.4 ± 657.6	7.3 ± 4.7	0.20 ± 0.12	41 ± 18
		150	Male	6.87 ± 1.64	1,330.3 ± 456.7	7.3 ± 3.0	0.36 ± 0.13	81 ± 14
			Female	11.00 ± 4.89	1,963.5 ± 1,273.1	8.8 ± 2.4	0.31 ± 0.19	73 ± 36

Data are presented as mean ± standard deviation. AUC_0-¥_, area under the curve from zero to infinity; C_max_, maximal serum concentration; MGAH22, chimeric anti-HER2 monoclonal antibody with an optimized Fc domain; RES120, chimeric anti-HER2 monoclonal antibody with wild-type human immunoglobulin G 1 Fc domain; T_1/2β_, elimination half-life; V_ss_, volume of distribution at steady state.

### Toxicology of MGAH22 in cynomolgus monkeys

MGAH22 recognizes cynomolgus HER2, and cross-reactivity studies with MGAH22 and trastuzumab revealed similar staining patterns against human and cynomolgus tissue panels (data not shown). Cynomolgus monkeys express activating and inhibitory FcγRs with substantially enhanced binding to the optimized Fc domain of MGAH22 relative to the WT Fc domain (Figure 2b). Although the increased binding to the inhibitory CD32B receptor could limit toxicity, it is compensated by the enhanced binding of the activating receptors, which is greater than that observed with human orthologs. When administered by intravenous infusion at a single 50 mg/kg dose or at 6 weekly doses of 15, 50, or 150 mg/kg, MGAH22 was well tolerated in male and female animals, and there were no clinical signs or treatment-related effects on food consumption, body weights, physical or ophthalmic examinations, blood pressure or heart rate, electrocardiogram, serum troponin I, hematology, serum chemistry, coagulation, or urine analysis parameters at any time during the study. There were no gross or microscopic findings at terminal necropsy attributable to MGAH22 administration and no findings in heart tissue.

Circulating NK cells (measured as CD3^-^/CD159a^+ ^cells by flow cytometry) were decreased, compared with pre-dose levels, by an average of 51% (range of 40% to 65%) in MGAH22-treated animals compared with 30% (range of 18% to 43%) in vehicle-treated animals within 1 day of dosing. Declines in NK cells were independent of dose, smaller in magnitude (less than 30%) after subsequent administrations, and not associated with changes in NK cell cytolytic activity when PBMCs from the treated monkeys were used as effector cells against K562 cells as targets (data not shown).

**Figure 6:**
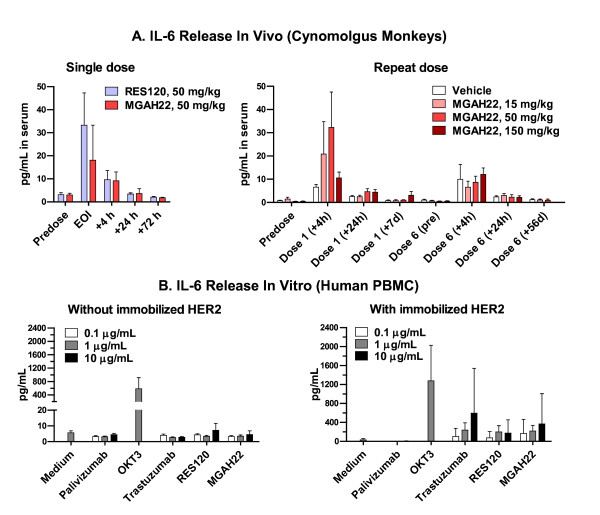
**MGAH22-induced IL-6 release *in vivo *and *in vitro***. **(a) **Serum IL-6 levels in single- and repeat-dose studies in cynomolgus monkeys. When MGAH22-treated groups were compared with RES120-treated groups (single-dose study) or with vehicle control group (repeat-dose study), there were no statistically significant changes in IL-6 levels. **(b) ***In vitro *IL-6 release from human peripheral blood mononuclear cells (PBMCs) incubated with the indicated antibodies on uncoated plates or plates coated with recombinant HER2 antigen. Statistically significant changes in IL-6 levels (*P *= 0.0313) were noted for MGAH22 compared with RES120 at concentrations of 1 or 10 μg/mL in the presence of immobilized HER2, but there were no significant differences between MGAH22 and trastuzumab. Data are presented as mean ± standard error of the mean. IL-6, interleukin-6; MGAH22, chimeric anti-HER2 monoclonal antibody with an optimized Fc domain; RES120, chimeric anti-HER2 monoclonal antibody with wild-type human immunoglobulin G 1 Fc domain.

Low levels of IL-6 (Figure 6a), but not IL-2, IL-4, IL-5, INF-γ, or TNF-α (data not shown), were transiently induced within 4 hours of infusion of MGAH22, RES120, or vehicle. From baseline levels of not more than 3 pg/mL, mean peak serum IL-6 levels after the first infusion were 11 to 32 pg/mL with RES120 or MGAH22 compared with 7 pg/mL with vehicle. After the sixth infusion in the repeat-dose study, mean peak serum IL-6 levels for all MGAH22 groups (7 to 12 pg/mL) were comparable to those in the vehicle group (10 pg/mL). Magnitude and duration of IL-6 induction by MGAH22 were dose-independent and comparable to those observed with RES120, indicating that cytokine release is not enhanced by the optimized Fc domain. This observation was corroborated by measuring antibody-induced cytokine release from human PBMCs in the presence or absence of immobilized HER2 antigen. The three anti-HER2 mAbs (MGAH22, RES120, and trastuzumab) induced similar levels of IL-6 (Figure 6b), TNF-α, and IFN-γ (data not shown) in the presence, but not absence, of HER2 antigen.

## Discussion

MGAH22 is a human/mouse chimeric IgG1 anti-HER2 antibody based on mouse clone 4D5, the precursor to trastuzumab. MGAH22 was engineered to maintain the antigen-binding properties of the original antibody while optimizing its interactions with human FcγRs, important mediators of antibody function *in vivo*. The engineered Fc domain of MGAH22 imparts increased affinity for both allelic variants of the low-affinity activating FcγR, CD16A, and decreased affinity for the inhibitory FcγR, CD32B. While maintaining the HER2-binding properties and direct anti-proliferative activity of trastuzumab against sensitive cell lines, these enhanced binding properties confer additional improvements in terms of enhanced anti-tumor activity against HER2-expressing tumor cell lines *in vitro*; the greatest improvement was observed in ADCC activity against the lower (1+ and 2+) HER2-expressing cell lines and/or with effector cells isolated from human donors homozygous or heterozygous for the low-binding allele (158F) of CD16A. *In vivo*, MGAH22 exhibited enhanced anti-tumor activity against a 2+ HER2-expressing cell line in mice genetically deficient for murine CD16 but transgenic for the human CD16A-158F variant.

The FcγR-binding profile of MGAH22 has many unique aspects. The increased binding affinity to CD16A compares well with that observed with afucosylated trastruzumab [36] or other Fc-engineered mAbs [37-39]. The improvement in MGAH22 binding to the 158F allele of human CD16A to levels exceeding those of the WT Fc domain for the 158V allele suggests that MGAH22 could provide benefit to patients of any CD16A genotype, but particularly homozygotes or heterozygotes carrying the 158F variant, who have poorer outcomes in response to trastuzumab treatment [11]. The increased CD16A binding also resulted in increased effectiveness, particularly against tumor cells expressing low levels of HER2. This suggests that MGAH22 can induce productive synapse formation between tumor and effector cells with fewer antibody-target interactions on the tumor cell surface, presumably by recruiting more Fc receptors on the effector cells per unit of binding or increasing the length of time the receptor is engaged or both. Because the benefit of trastuzumab therapy accrues only to patients with tumors that overexpress HER2 at the 3+ level or exhibit gene amplification [3,5,40], this finding suggests that MGAH22 may extend such advantages of anti-HER2 therapy to patients whose tumors express low HER2 levels and who are not thought to benefit from trastuzumab treatment.

CD16A is coexpressed with other FcγRs on mononuclear phagocytes but is the only FcγR expressed by NK cells. These cells are the major contributors to ADCC activity in PBMCs under standard *in vitro *assay conditions, a notion supported by the observation that enhanced MGAH22-mediated ADCC was also observed with purified NK cells. NK cells have been implicated as important mediators of the anti-tumor activity of trastuzumab in patients with breast cancer. Trastuzumab treatment is associated with increased numbers of tumor-associated NK cells, and patients with responsive tumors tend to have larger numbers of tumor-infiltrating NK cells [8,9]. Patients with higher NK cell numbers exhibit higher levels of trastuzumab-mediated ADCC activity, which has been associated with increased tumor responsiveness [9,10]. This finding is consistent with the association between responsiveness to trastuzumab treatment and level of ADCC activity mediated by CD16-expressing cells and CD16A genotype [11].

Another unique feature of the Fc domain of MGAH22 is its decreased binding to the CD32B inhibitory receptor. Fc domains exhibiting decreased fucosylation, by comparison, enhance binding only to CD16A [39,41], and other mutations reported to increase binding to activating receptors also demonstrate increased binding to CD32B but to different extents, depending on the mutations [37,38]. When co-engaged with an activating FcγR on mononuclear phagocyte effectors, CD32B confers an inhibitory signal that counters cell activation. Although no clinical data on an association between reduced CD32B binding and response to trastuzumab or other mAbs are available, non-clinical studies show the importance of Fc-mediated functions exerted by monocytes and macrophages *in vivo *[42]. Enhanced anti-tumor responses occur in mice genetically lacking CD32B [7], and enhanced antigen delivery via immune complexes that bind both activating and inhibitory receptors occurs under conditions of CD32B blockade [43-45]. These effects may contribute to the ability of immunotherapy to break tolerance in cancer and induce an adaptive immune response. Attempts at modeling the CD32B-dependent component of MGAH22 action in terms of effector cell function have been hampered by the ineffective tumor cytotoxic activity of mononuclear phagocytes *in vitro *(data not shown) and lack of a suitable animal model. Nonetheless, a decline in binding to CD32B is expected to be beneficial by increasing the ratio of activating-to-inhibitory FcγR interactions.

In the selection of species for non-human primate toxicology studies, both antigen expression and Fc/FcγR interactions were considered. Tissue cross-reactivity studies with MGAH22 on human and cynomolgus tissue panels revealed similar antigen distributions, which were comparable to those observed with trastuzumab. Importantly, the binding profile of MGAH22 to cynomolgus monkey FcγRs generally supports the use of this species as a relevant toxicology model for the immune effector function of this antibody. Although the engineered Fc domain has increased binding to cynomolgus monkey CD32B, which differs from its decreased binding to human CD32B and may limit toxicity in monkeys, it has increased binding to the invariant monkey CD16A and CD32A receptors. Moreover, the binding affinities for these activating FcγRs of monkeys exceed those for the high-binding alleles of the human orthologs, a situation that may counteract the potential inhibitory effect of increased binding to monkey CD32B and be adequate for evaluating potential toxic effects due to FcγR engagement.

The MGAH22 Fc domain preserves FcRn binding, which favors an extended serum half-life [46]. The terminal half-life of MGAH22 in cynomolgus was 7 to 9 days, approximately 20% shorter than that of RES120, which contains the WT Fc domain. A similar decline in half-life was observed when afucosylated trastuzumab, exhibiting increased binding to hCD16A, was compared with trastuzumab in hCD16A transgenic mice [36]. Except for a slightly shorter half-life, the pharmacokinetic profile of MGAH22 in cynomolgus monkeys is comparable to that of other anti-HER2 mAbs [34,35]. Importantly, in the single- and repeat-dose toxicology studies, there were no significant antibody-related clinical observations or macro/microscopic findings. The modest dose-independent decrease in circulating NK cells was reminiscent of a similar observation in monkeys treated with an Fc domain-enhanced anti-CD19 mAb [37]. Given its transient nature, the phenomenon likely results from margination of the NK cells. The Fc-engineered MGAH22 mAb was not unusually immunogenic in monkeys, but owing to the lack of predictive value of immunogenicity data in animals [47], the potential incidence of immunogenicity in humans cannot be extrapolated.

Cytokine release could be exacerbated by increased binding to FcγRs. MGAH22 induced minimal levels of just IL-6 in cynomolgus monkeys and IL-6, TNF-α, and IFN-γ from human PBMCs *in vitro *that were similar to those induced by RES120 or trastuzumab, suggesting that MGAH22 is unlikely to induce cytokines in patients to levels any higher than those induced by trastuzumab. A potential explanation is that cytokine release may relate more to CD32A than CD16A. CD32A expression by mononuclear phagocytes, but not NK cells, is consistent with the spectrum of observed cytokines, which did not include IL-2, an NK cell-derived cytokine. Binding of MGAH22 to the prevalent CD32A-131H allele is unchanged compared with WT Fc domains, whereas binding to the rarer CD32A-131R allele is decreased, a reflection of the high degree of homology between the extracellular domain and Fc-binding interface of this variant with CD32B (including the arginine at position 131, which is shared by CD32B). While CD32A polymorphism may contribute to outcomes in patients with trastuzumab-treated metastatic breast cancer, its role is less pronounced than that associated with CD16A polymorphism [11]. Moreover, recent data suggest an association of the 131H allele of CD32A with the development of trastuzumab-related cardiotoxicity [48]. Thus, the lack of enhanced binding to either of the CD32A alleles may be favorable to the safety profile of MGAH22.

Other novel HER2-directed agents are undergoing clinical development. A trastuzumab-drug conjugate, T-DM1, designed to deliver a cytotoxic molecule into HER2-overexpressing cells via receptor-mediated endocytosis [49], has shown a significant advantage in advanced breast cancer, although its benefits appear to be restricted to patients with HER2 3+ or gene-amplified tumors [50]. In this context, MGAH22 may have particular utility in patients with low HER2-expressing tumors. An afucosylated version of trastuzumab with increased anti-tumor effector function has also been described [36]. However, MGAH22, by exhibiting diminished binding to the inhibitory FcγR, CD32B, differs from afucosylated trastuzumab, which exhibits a slight increase in binding to this inhibitory receptor. MGAH22, by diminishing interactions with this inhibitory FcγR, would be expected to exhibit additional favorable properties in the presence of mononuclear phagocytic effector cells and potentially further enhanced efficacy against low HER2-expressing tumors or tumors resistant to trastuzumab therapy.

## Conclusions

The favorable safety profile of MGAH22 is reflected by its 'no observed adverse effect level' (NOAEL) in cynomolgus monkeys of 150 mg/kg. The minimal effective human equivalent dose is approximately 0.1 mg/kg, estimated from a minimum effective dose of 1 mg/kg in xenograft models with human CD16A transgenic mice (10-fold lower than that of the corresponding WT mAb). Based on these considerations, a phase 1 dose-escalation study with MGAH22 doses ranging from 0.1 to 15 mg/kg has been initiated in patients with HER2-expressing tumors.

## Abbreviations

ADA: anti-drug antibody; ADCC: antibody-dependent cell-mediated cytotoxicity; CBA: cytometric bead array; ch4D5-N297Q: chimeric anti-HER2 monoclonal antibody with inactivated Fc domain; EC_50_: effective concentration for 50% lysis or 50% binding; FcγR: Fc-gamma receptor; IFN-γ: interferon-gamma; IgG1: immunoglobulin G 1; IL: interleukin; K_D_: equilibrium dissociation constant; mAb: monoclonal antibody; MGAH22: chimeric anti-HER2 monoclonal antibody with an optimized Fc domain; MGFc0264: human IgG1 Fc domain optimized for increased CD16A and decreased CD32B binding; NK: natural killer; PBMC: peripheral blood mononuclear cell; RES120: chimeric anti-HER2 monoclonal antibody with wild-type human immunoglobulin G 1 Fc domain; TNF-α: tumor necrosis factor-alpha; WT: wild-type.

## Competing interests

All authors are or have been employed by MacroGenics, Inc., a privately held company, and have received MacroGenics stock options as a condition of employment.

## Authors' contributions

SG, WZ, YY, HL, SB, LH, VC, and TZ conducted experiments and helped to analyze data. SJ, PAM, JS and SK helped to conceive and design experiments and analyze data. SJS helped to analyze data. EB and JLN helped to conceive and design experiments, analyze data, and write the paper. All authors read and approved the final manuscript.
